# A reproducible optical–microscopic framework for evaluating electronic apex locator performance in different irrigants using 3D-printed transparent resin teeth

**DOI:** 10.3389/fdmed.2026.1839221

**Published:** 2026-05-25

**Authors:** Tran-Lan-Khue Pham, Quoc-Viet Lam, Dai-Phong Lam, Thi-Nguyet-Anh Nguyen, Hoang-Vinh Le, Thi-Thuy-Trang Huynh, Huynh-Anh Bui, Hoang-Lan-Anh Le, Phuong-Doan Phan, Nguyen-Tra-Mi Le, Thuan-Loc Tran, An-Tran Pham, Trieu-Khang Pham, Ngoc-Phuong-Anh Nguyen, Van-Khoa Pham, Anh-Minh Ngo, Thi-Kim-Nguyen Vo, Thi-Bich-Van Tran, Ngoc-Phuc Nguyen

**Affiliations:** 1 Faculty of Dentistry, University of Medicine and Pharmacy at Ho Chi Minh City, Ho Chi Minh City, Vietnam; 2 Faculty of Dentistry, Hong Bang International University, Ho Chi Minh City, Vietnam; 3 Private Practice, Ho Chi Minh City, Vietnam

**Keywords:** apical foramen, electronic apex locator, endodontic working length, root canal length, transparent resin tooth

## Abstract

This study presents a standardized experimental framework for assessing the different modes of locating the apical foramen in EAL-integrated endodontic motors using 3D-printed transparent resin teeth in two electrolyte solutions. Ten extracted human premolars were scanned using cone-beam computed tomography and replicated to produce 100 transparent resin tooth models. Root canal length determination was performed under static and dynamic conditions using two EAL-integrated endodontic motors in sodium chloride and chlorhexidine solutions. A dual optical microscope system enabled micron-level positional recording of the file tip relative to the apical terminus, independent of the electronic readout. Extracted human teeth served as reference specimens for validation. High agreement was observed among different operating modes of the endodontic motors, with no significant differences between irrigation solutions. Measurements demonstrated high repeatability and consistency across static and dynamic conditions. The proposed framework enables direct, objective, and reproducible evaluation of EAL performance by combining anatomical standardization with high-precision optical measurement. Transparent resin teeth allow continuous visualization of file behavior, facilitating assessment of dynamic motor functions that are difficult to quantify using conventional approaches. This framework provides a scalable platform for preclinical device evaluation, methodological validation, and simulation-based endodontic education.

## Introduction

Accurate determination of the root canal length is a critical prerequisite for successful endodontic treatment and remains a core learning objective in preclinical endodontic education. Electronic apex locators (EALs) are widely accepted as reliable adjuncts to radiographic methods, owing to their ability to identify the apical terminus based on impedance changes within the root canal system (1–3). Contemporary EALs are increasingly integrated into endodontic motors, enabling root canal length determination to be performed under both static and dynamic instrumentation conditions (4, 5).

Despite their widespread clinical use, the methodological evaluation of EAL performance remains challenging. *Ex vivo* studies have traditionally relied on extracted human teeth as reference specimens because of their anatomical authenticity (1, 3, 6–8). However, the use of natural teeth is constrained by limited availability, ethical and biosafety considerations, and substantial anatomical variability, particularly in apical morphology (9). These factors reduce experimental reproducibility and complicate objective comparison among devices, operating modes, and experimental conditions.

Recent advances in cone-beam computed tomography (CBCT) and three-dimensional (3D) printing have enabled the fabrication of anatomically standardized tooth models derived from natural dentition. Such models provide improved experimental control while preserving clinically relevant canal morphology (3, 6–8). In endodontic research, resin-based tooth replicas have been increasingly used to reduce biological variability and enhance measurement consistency (9–11). The resin-based tooth model could be used successfully for root canal length measurement because it is non-conductive, which is similar to that of a natural tooth, acting as a capacitor (12). Most commercially available resin teeth are opaque, limiting direct visualization of file position within the canal and requiring indirect measurement approaches similar to those used with extracted teeth, resulting in difficulty in evaluating the dynamic modes of EAL-integrated endodontic motors.

Transparent 3D-printed resin teeth represent a promising alternative for both methodological research and educational simulation. Their optical clarity allows direct observation of instrument advancement and apical approach, which may be advantageous for evaluating dynamic EAL functions such as auto-stop and auto-reverse mechanisms. Although previous studies have shown that resin materials can approximate the electrical behavior of dental hard tissues under controlled conditions (13–16), the application of transparent resin teeth for systematic evaluation of EAL-integrated endodontic motors has not been comprehensively investigated. Besides the blindness situation in almost every setting-up, there is a lack of a direct measurement method for evaluating the distance between other positions investigated in the previous studies (3, 6–8).

Beyond research applications, standardized 3D-printed tooth models have gained increasing attention in preclinical endodontic education. Recent systematic reviews and comparative studies have demonstrated that simulation-based training using artificial teeth can improve conceptual understanding, learning consistency, and assessment reliability when compared with traditional extracted tooth models (15, 17, 18). In particular, models that allow visual feedback of internal canal anatomy may facilitate a deeper understanding of abstract concepts such as apical constriction location and electronic root canal length determination. Recent studies have shown that the root canal length measurements were taken by using a reference point on the occlusal surface, which could not be exactly the same at different times of measurement, that could cause inaccurate outcomes (3, 7).

Therefore, the aim of the present *in vitro* study was to develop and validate a reproducible experimental framework for evaluating the reliability, repeatability, and precision of different modes of locating the apical foramen in EAL-integrated endodontic motors using transparent 3D-printed resin teeth in the two electrolyte solutions. By combining anatomical standardization with proper measurements under static and dynamic conditions in different irrigation environments, this study seeks to address existing methodological gaps while supporting the continued development of robust simulation-based approaches for preclinical research and education.

## Materials and methods

### Study design

This *in vitro* experimental study evaluated EAL-integrated endodontic motors using standardized, transparent resin tooth models under controlled laboratory conditions.

### Ethical considerations

The use of extracted human teeth was approved by the institutional review board of the authors' institution (approval number: 1043/HĐĐĐ-ĐHYD). The informed consent was obtained from the parents of all participants, or the participants themselves, confirming that they would provide their own extracted teeth for the research. All procedures performed in the study involving human participants were in accordance with the ethical standards of the institutional and/or national research committee and with the 1964 Helsinki declaration and its later amendments or comparable ethical standards.

### Sample size calculation

The sample size was calculated using the statistical software version 23.4.5 (MedCalc Software, Ostend, Belgium) for the sample size function for Bland–Altman plot with a type I error of 0.05 and a type II error of 0.20, expected mean of differences and maximum allowed differences between methods were 0.1 and 0.5, respectively, resulted in 10 samples.

### Tooth selection and preparation

Ten extracted human premolars with single canals and mature apices were selected. These teeth were extracted by the reasons of orthodontic treatment or periodontal disease. Teeth with fractures, resorption, or previous endodontic treatment were excluded. Specimens were cleaned and stored in 0.1% thymol solution at 4 °C until use. The tooth with the apical foramen at the outline of the root, which could be seen when observed from the profile view that facilitating the determination of the apical terminus of the transparent resin tooth replica in the clear electrolyte solutions.

Standardized access cavities were designed by proper tools in the software after CBCT acquisition and were available right after produced.

### CBCT acquisition and resin model fabrication

All teeth were scanned using a CBCT system (Veraview X800, J. Morita, Japan) with the parameters: kVp of 98.0, mA of 8.0, voxel size of 0.08 mm, slice thickness of 0.16 mm, exposure time of 9.4 s, FOV of 40.4 mm × 40.4 mm × 40.16 mm. The CBCT data were exported in Digital Imaging and Communications in Medicine (DICOM) format. Segmentation of the tooth structures was performed using 3D Slicer software, version 5.8. The DICOM data were first imported into the software environment, after which the Segment Editor module was activated to create a new segmentation label. The boundaries between dental tissue and surrounding structures were identified using the Level Tracing tool. Segmentation was performed progressively from the crown toward the root apex to ensure accurate delineation of the tooth anatomy. Areas presenting segmentation errors or undesired attachment to surrounding bone structures were corrected using the Erase and Scissors tools. After completion of segmentation, a Gaussian smoothing algorithm was applied within the Smoothing module to improve the surface quality while preserving anatomical details, particularly in the apical region.

The segmented tooth model was then exported as an STL file and imported into Meshmixer [Meshmixer, version 3.5, (Autodesk, US).software] for digital access cavity preparation. The Inspector tool was first applied to detect and automatically repair potential mesh defects on the surface of the model. To simulate the access cavity, a primitive geometric object was inserted from the Meshmix library. For premolars, an oval cylindrical shape was used to reproduce the typical morphology of the access cavity. The primitive object was positioned at the intended access site and adjusted in orientation, size, and inclination using the Transform function to align with the long axis and anatomical features of the tooth. A Boolean Difference operation was then applied by selecting the tooth model as the base object and the primitive as the subtractive element, thereby removing the intersecting volume and creating the simulated access cavity. After the Boolean operation, the edges of the cavity were refined using the Sculpt Smooth tool to eliminate sharp irregularities and produce a smoother internal surface. The final modified model was subsequently exported again in STL format for the next stage of the workflow.

The STL file was then imported into Chitubox software (version 1.1, CBD-Tech, China) for preparation for the three-dimensional printing process. Within the software environment, the model was inspected to verify morphological integrity, dimensional accuracy, and surface continuity in order to detect potential defects that could affect printing quality. The model was then oriented within the printing space to optimize printing accuracy, minimize the contact area with the build platform, and reduce potential distortion in critical anatomical regions. Support structures were automatically generated and manually adjusted to ensure sufficient stabilization of the model during printing while avoiding placement in areas where surface details needed to be preserved. The model was subsequently sliced using the slicing function of the software, during which appropriate printing parameters such as layer thickness and exposure settings were configured according to the printer specifications. The final output file in CTB format was then transferred to the 3D printer for fabrication.

Ten physical replicas were fabricated from the digitized 3D model of each natural tooth using a 3D printer (Saturn Ultra 3, Elegoo, China) with the layer thickness of photopolymer resin was 0.05 mm (Standard Plus Resin High Clear, JAMG HE, Shenzhen Yongchanghe Technology, China). Printing parameters and post-curing conditions were standardized for all models.

### Mounting system and irrigation environment

Each resin tooth was mounted in a custom-designed transparent resin container filled with either 0.9% saline solution (0.9% Sodium Chloride Intravenous Infusion, B. Braun, Hanoi, Vietnam) or 2% chlorhexidine digluconate (GLUCO-CheX 2%, Cerkamed, Stalowa Wola, Poland). A partial resin cover ensured stable and reproducible positioning during measurements.

### Experimental setup

**Figure 1:**
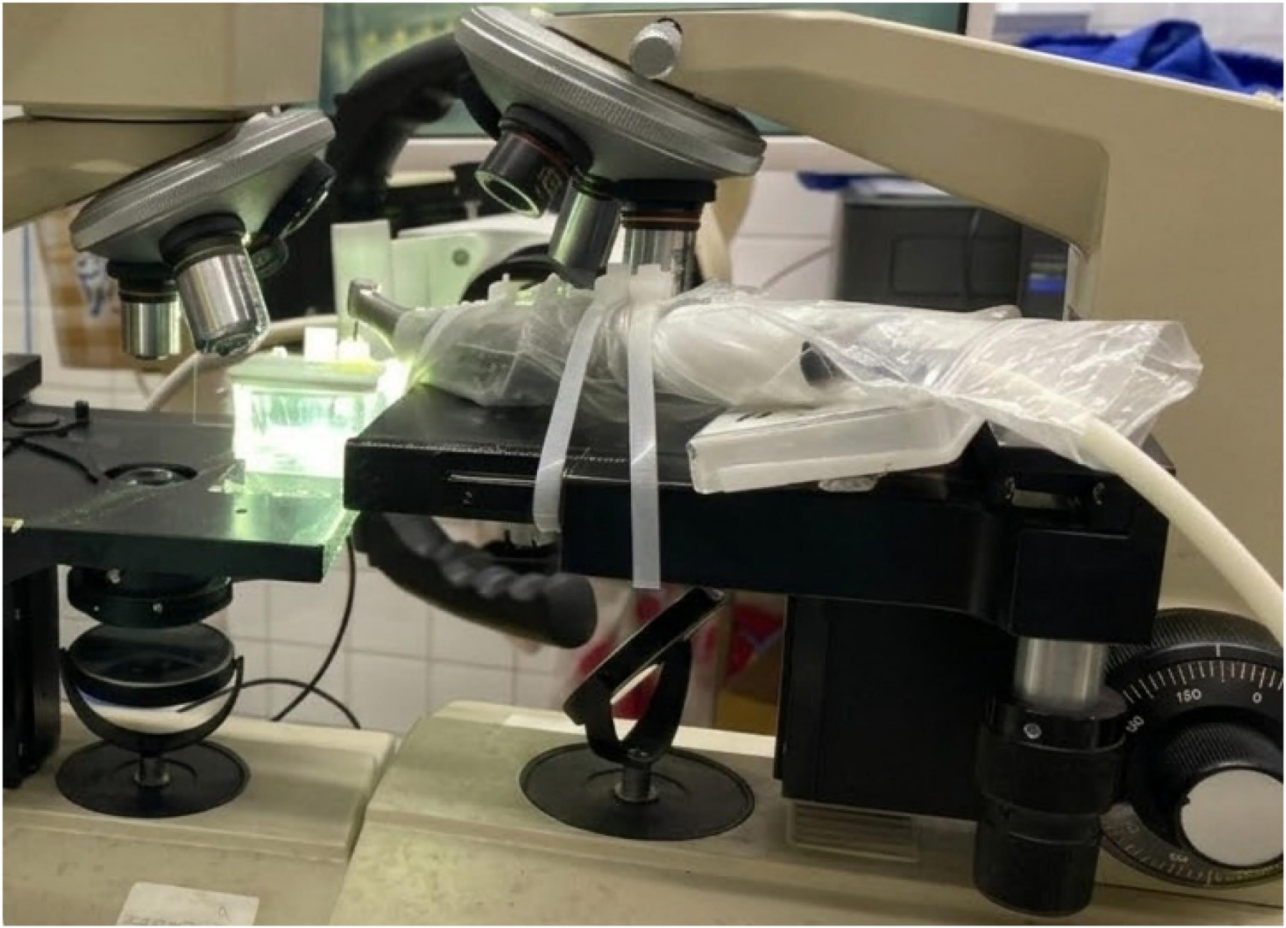
Dual optical microscope framework for root canal length measurement.

**Figure 2:**
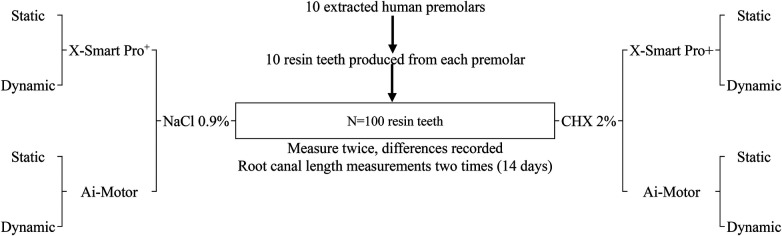
Flowchart of the study.

A dual optical microscope system was employed. One plain stage of a microscope fixed the handpiece of the endodontic motor, allowing controlled vertical movement via a fine adjustment knob with micron-level resolution at graduated increments of 2.5 µm. Another plain stage of the second microscope supported the resin container and enabled specimen replacement using the coarse adjustment knob (Figures 1, 2). All procedures were recorded using a 3D operating microscope (Seiler Medical, St. Louis, MO, USA).

### Endodontic motors and instruments

Two EAL-integrated endodontic motors were evaluated: X-Smart Pro^+^ (Dentsply Sirona, Ballaigues, Switzerland) and an Ai-Motor (Gullin Woodpecker Medical Instrument, Guangxi, China). WaveOne Gold Small files (size 20, taper 0.07; Dentsply Sirona, Ballaigues, Switzerland) were used following the manufacturer's instructions. New files were used for each specimen.

### Root canal length determination

Measurements were performed under static and dynamic conditions. First, in static mode, the file was advanced without rotation until the apex was indicated by the EAL. Then, in dynamic mode, the file was operated in reciprocating motion according to the manufacturer's settings.

For the X-Smart Pro^+^, measurements were obtained in standstill, standstill with file-clip, auto-stop, and auto-reverse modes. For the Ai-Motor, static, auto-stop, and auto-reverse modes were evaluated. The position of the file tip at apical indication was recorded using the fine adjustment scale of the optical microscope.

### Reproducibility and blinding

Each measurement was repeated twice per condition. All measurements were performed by a calibrated operator and repeated after a 2-week interval to assess intra-operator repeatability. Data analysis was conducted by an independent investigator blinded to device type and measurement mode.

### Statistical analysis

Data distribution was assessed using the Shapiro–Wilk test. Agreement and repeatability were analyzed using Bland–Altman plots with statistical software (MedCalc Software, Ostend, Belgium). Statistical significance was set at 0.05.

## Results

A high level of agreement was observed among the different operating modes of the endodontic motors across both irrigation solutions. All measurements of the root canal length for groups of resin model teeth were checked and showed that they have normally distributed. The Intraclass Correlation Coefficient (ICC) was also greater than 0.8 for all repeatabilities.

Table 1 presents the Bland–Altman analysis parameters assessing agreement among the apex locator modes within each motor system in both irrigation solutions. Overall, minimal disagreement was observed between the standstill mode and other operating modes of the X-Smart Pro^+^ motor in both solutions, with the exception of the standstill mode combined with the file-clip configuration in chlorhexidine. In addition, discrepancies were identified between the static and dynamic modes in both irrigants. For the Ai-Motor, the auto-stop mode demonstrated agreement with the auto-reverse mode in chlorhexidine and with the standstill mode in sodium chloride, suggesting mode-dependent variability influenced by the irrigant.

**Table 1:** The agreements between the modes of apex locators in the two solutions with the transparent resin tooth models.

Motor	Mode	Solution	Arithmetic mean	LLoA	95% CI of LLoA	ULoA	95% CI of ULoA	CR	*P*		Bias	
									Regression	Paired *t*-test	Proportional	Fixed
XS	AS vs. AR	NaCl	0.9600	−24.4551	−28.8668 to −20.0433	26.3751	21.9633–30.7868	25.3576	0.0530	0.4608	No	No
		CHX	0.3000	−15.0410	−17.7040 to −12.3780	15.6410	12.9780–18.3040	15.2754	0.2685	0.7023	No	No
	AS vs. S	NaCl	3.3900	−22.0281	−26.4404 to −17.6158	28.8081	24.3958–33.2204	26.1489	0.0024*	0.0103*	Yes	Yes
		CHX	2.2000	−13.9385	−16.7400 to −11.1371	18.3385	15.5371–21.1400	16.6265	<0.0001*	0.0088*	Yes	Yes
	AR vs. S	NaCl	3.3900	−22.0281	−26.4404 to −17.6158	28.8081	24.3958–33.2204	26.1489	0.0024*	0.0103*	Yes	Yes
		CHX	1.9000	−14.2686	−17.0753 to −11.4619	18.0686	15.2619–20.8753	16.5129	<0.0001*	0.0234*	Yes	Yes
	AS vs. SFC	NaCl	0.3600	−30.3050	−35.6281 to −24.9819	31.0250	25.7019–36.3481	30.5194	0.0410*	0.8185	Yes	No
		CHX	0.9200	−15.8081	−18.7119 to −12.9043	17.6481	14.7443–20.5519	16.7417	0.5721	0.2837	No	No
	AR vs. SFC	NaCl	−0.6000	−28.4290	−33.2598 to −23.5982	27.2290	22.3982–32.0598	27.7144	0.7816	0.6735	No	No
		CHX	0.6200	−15.6282	−18.4488 to −12.8077	16.8682	14.0477–19.6888	16.2124	0.6195	0.4563	No	No
	S vs. SFC	NaCl	−3.9900	−27.3884	−31.4501 to −23.3267	19.4084	15.3467–23.4701	24.5595	0.0049*	0.0012*	Yes	Yes
		CHX	−1.2800	−17.9347	−20.8257 to −15.0436	15.3747	12.4836–18.2657	16.7600	<0.0001*	0.1352	Yes	No
AI	AS vs. AR	NaCl	3.1900	−21.9977	−26.3701 to −17.6254	28.3777	24.0054–32.7501	25.8296	0.8515	0.0147	No	Yes
		CHX	1.6300	−15.9464	−18.9975 to −12.8954	19.2064	16.1554–22.2575	17.7778	0.7493	0.0721	No	No
	AS vs. S	NaCl	3.4700	−19.0719	−22.9849 to −15.1588	26.0119	22.0988–29.9249	23.4374	<0.0001*	0.0032*	Yes	Yes
		CHX	3.5200	−13.1286	−16.0186 to −10.2386	20.1686	17.2786–23.0586	17.9444	<0.0001*	0.0001*	Yes	Yes
	AR vs. S	NaCl	0.2800	−22.1663	−26.0628 to −18.2699	22.7263	18.8299–26.6228	22.3406	<0.0001*	0.8074	Yes	No
		CHX	1.8900	−15.7245	−18.7821 to −12.6668	19.5045	16.4468–22.5621	17.9134	<0.0001*	0.0380*	Yes	Yes

XS, X-Smart Pro^+^ motor; AI, Ai-motor; LLoA, lower limit of agreement; ULoA, upper limit of agreement; AS, auto stop; AR, auto reverse; CHX, chlorhexidine; CI, confidence interval; S, standstill; SFC, standstill with file-clip; CR, coefficient of repeatability; vs., versus.

* *P* < 0.05.

Table 2 summarizes the agreement between corresponding operating modes of the same endodontic motor when used in sodium chloride and chlorhexidine solutions. Several agreements were observed across almost all apical locating modes for each motor, although some proportional biases were detected in certain comparisons. These findings show that the type of irrigating solution did not substantially influence the performance of electronic apex locators across different operating modes.

**Table 2:** The agreements between modes of the same endodontic motor in the two solutions.

Motor	Solution	Mode	Arithmetic mean	LLoA	95% CI of LLoA	ULoA	95% CI of ULoA	CR	*P*		Bias	
									Regression	Paired *t*-test	Proportional	Fixed
XS	NaCl vs. CHX	AS	1.4800	−28.2559	−33.4178 to −23.0941	31.2159	26.0541–36.3778	29.7287	<0.0001*	0.3317	Yes	No
		AR	0.8200	−22.7583	−26.8512 to −18.6654	24.3983	20.3054–28.4912	23.5151	<0.0001*	0.4971	Yes	No
		S	−0.6700	−19.3870	−22.6361 to −16.1380	18.0470	14.7980–21.2961	18.6695	<0.0001*	0.4846	Yes	No
		SFC	2.0400	−25.2112	−29.9417 to −20.4807	29.2912	24.5607–34.0217	27.4078	0.0002*	0.1455	Yes	No
AI	NaCl vs. CHX	AS	−0.4000	−25.6221	−30.0004 to −21.2438	24.8221	20.4438–29.2004	25.1079	0.0024*	0.7566	Yes	No
		AR	−1.9600	−28.6297	−33.2592 to −24.0001	24.7097	20.0801–29.3392	26.8126	0.0106*	0.1529	Yes	No
		S	−0.3500	−16.0186	−18.7384 to −13.2987	15.3186	12.5987–18.0384	15.6051	<0.0001*	0.6625	Yes	No

XS, X-Smart pro^+^ motor; AI, Ai-Motor; LLoA, lower limit of agreement; ULoA, upper limit of agreement; AS, auto stop; AR, auto reverse; CHX, chlorhexidine; CI, confidence interval; S, standstill; SFC, standstill with file-clip; CR, coefficient of repeatability; vs., versus.

* *P* < 0.05.

Table 3 illustrates the agreement between the two endodontic motors when operated under identical modes in both irrigation solutions. A single disagreement was noted between the two motors in the auto-reverse mode when measurements were performed in sodium chloride. All other operating modes demonstrated good agreement between the two devices in both solutions. These results suggest that the two endodontic motors may be used interchangeably for root canal length determination under several operating modes in chlorhexidine and sodium chloride, but the auto-reverse mode in sodium chloride solution.

**Table 3:** The agreements between the two endodontic motors in the same modes in the two solutions.

Motor	Solution	Mode	Arithmetic mean	LLoA	95% CI of LLoA	ULoA	95% CI of ULoA	CR	*P*		Bias	
									Regression	Paired *t*-test	Proportional	Fixed
XS vs. AI	NaCl	AS	1.4100	−32.1682	37.9970 to −26.3394	34.9882	29.1594–40.8170	33.5240	0.1152	0.4125	No	No
		AR	3.6400	−24.0471	−28.8533 to −19.2409	31.3271	26.5209–36.1333	28.4571	0.9995	0.0114*	No	Yes
		S	0.5300	−19.6542	−23.1579 to −16.1504	20.7142	17.2104–24.2179	20.1098	0.0264*	0.6079	Yes	No
	CHX	AS	−0.4700	−20.8836	−24.4271 to −17.3400	19.9436	16.4000–23.4871	20.3321	0.5427	0.6528	No	No
		AR	0.8600	−18.9938	−22.4402 to −15.5474	20.7138	17.2674–24.1602	19.8261	0.0568	0.3979	No	No
		S	0.8500	−10.5323	−12.5082 to −8.5565	12.2323	10.2565–14.2082	11.4471	0.5796	0.1465	No	No

XS, X-Smart Pro^+^ motor; AI, Ai-Motor; LLoA, lower limit of agreement; ULoA, upper limit of agreement; AS, auto-stop; AR, auto-reverse; CHX, chlorhexidine; CI, confidence interval; S, standstill; SFC, standstill with file-clip; vs., versus.

* *P* < 0.05.

## Discussion

The present study introduces a reproducible optical–microscopic framework for evaluating the performance of electronic apex locator (EAL)–integrated endodontic motors using 3D-printed transparent resin teeth. When interpreted in the comprehensive literature, this approach addresses several methodological limitations associated with previously used experimental models and offers added value for both preclinical research and endodontic education (3, 5, 7, 19).

The present *in vitro* study demonstrated a high level of agreement among different operating modes of two contemporary endodontic motors when used with 0.9% sodium chloride (3, 5, 7, 19) and 2% chlorhexidine. These findings suggest that the tested irrigants exert minimal influence on the performance of electronic apex locator (EAL) functions embedded in modern endodontic motors, regardless of static or dynamic measurement conditions.

The observed consistency between irrigants aligns with recent studies reporting stable EAL precision in electrically conductive solutions such as saline and chlorhexidine, which provide favorable conditions for impedance-based measurements without interfering with apical signal detection. The complete agreement between the operating modes of the same motor across both solutions further supports the assumption that irrigant composition, within the tested range, does not significantly alter apical positioning outcomes.

Agreement between the two motors across most operating modes indicates comparable technological reliability between the Ai-Motor (Woodpecker) and X-Smart Pro+. The isolated disagreement observed in the auto-reverse mode in sodium chloride may reflect differences in torque-control algorithms or mode-specific signal interpretation rather than a true irrigant-related effect. These two EALs were chosen because of their accuracy and reliability. However, the Ai-Motor has no function of SFC, whereas the X-Smart Pro + could use the clipped file in the standstill mode to measure the root canal length.

Clinically, these findings suggest that both motors may be used interchangeably for root canal length determination in saline or chlorhexidine environments, offering flexibility in clinical decision-making. However, mode-specific behavior, particularly in auto-reverse functions, should be interpreted cautiously.

Extracted human teeth have historically been regarded as the reference standard for evaluating EAL accuracy. Foundational and contemporary studies have shown that modern EALs can determine root canal length with acceptable accuracy under controlled conditions (1, 3, 6–8). However, most of these investigations relied on indirect assessment methods, including radiographic evaluation, visual inspection after tooth sectioning, or subtraction of file length relative to the apical foramen. Such approaches are inherently limited by operator dependency, restricted spatial resolution, and reduced reproducibility. In addition, the substantial biological variability among extracted teeth, such as differences in canal diameter, apical constriction morphology, dentin conductivity, and tissue hydration-has been widely recognized as a confounding factor that complicates comparative analysis and limits experimental standardization (9).

To overcome some of these limitations, acrylic blocks with simulated canals have been widely used, particularly in preclinical endodontic education. Acrylic models offer excellent standardization and allow direct visualization of instrument motion, making them suitable for teaching canal shaping and instrumentation techniques (10, 15, 17). The electrical properties of acrylic materials differ markedly from those of natural dental tissues, limiting their validity for evaluating impedance-based devices such as EALs. While acrylic blocks are valuable educational tools, their applicability for investigating electronic root canal length determination remains limited (17).

Using transparent 3D-printed resin teeth in the present study represents a methodological advancement over previously described resin models. By combining CBCT-derived anatomical fidelity with full visual access to the internal canal system, transparent resin teeth enable continuous observation of file progression and apical approach. This feature is advantageous for evaluating dynamic EAL functions, such as auto-stop and auto-reverse modes, which depend not only on electronic detection but also on the timing and motion of the rotating instrument. Despite the widespread clinical use of motor-driven instrumentation, dynamic root canal length determination remains underrepresented in the EAL literature, which has traditionally focused on static measurement conditions (3, 4, 6–8, 16).

Another key contribution of the present framework lies in integrating dual optical microscopy for direct positional measurement. Unlike conventional approaches that infer file position from electronic readouts or post-experimental measurements, the optical–microscopic system enables micron-level recording of file tip displacement relative to the apical terminus (3, 5, 7, 19). By decoupling physical position measurement from electronic indication, this approach reduces operator bias and enhances measurement traceability.

Beyond its research implications, the proposed framework has important relevance for preclinical endodontic education. One of the persistent challenges in teaching root canal length determination lies in conveying the abstract relationship between electronic readings and apical anatomy. Transparent resin teeth allow students to directly visualize file position relative to the apex while simultaneously observing EAL feedback. This immediate visual correlation may enhance conceptual understanding and reduce reliance on trial-and-error learning, consistent with evidence supporting simulation-based and standardized teaching models in endodontic education and training.

Using standardized 3D-printed models facilitates consistent training experiences across student cohorts. Unlike extracted teeth, which vary widely in anatomy and condition, resin models allow educators to control case complexity and progressively introduce anatomical challenges. This standardization also enables more objective assessment of student performance and may improve the reliability of preclinical examinations.

Recent studies show saline or chlorhexidine produce more stable apex-locator readings than NaOCl. NaOCl is a powerful irrigant (strongly antimicrobial and tissue-dissolving) but its high ionic conductivity can distort electronic measurements (20). In bench tests, saline's neutral conductivity and CHX's relatively low conductivity yield more accurate EAL performance (21, 22). Saline is biocompatible and inert (no disinfection or tissue dissolution), while CHX adds antimicrobial substantivity without corroding instruments. NaOCl (0.5%–5.25%) often shortens working length readings and adds variability (20, 22). Clinically NaOCl is essential for cleaning, but for testing EALs practitioners prefer saline or CHX to avoid NaOCl's interference. Irrigating with saline or CHX while verifying motor-integrated electronic apex locators yields consistent, reproducible measurements, and with the availability, odorless, and low-cost of saline, practical endodontic education has certain helpful aspects.

Several limitations of the present framework should be acknowledged. Resin materials cannot fully replicate the microstructural complexity, fluid dynamics, and biological variability of natural dentin and periodontal tissues. Besides these issues, resin aging, optical distortion, irrigant absorption, printing resolution, or reproducibility between print batches are also important factors in the present study. The framework is not intended to replace clinical or *in vivo* evaluation but to serve as a controlled platform for preclinical assessment. In addition, the present study focused on single-rooted premolars with relatively simple canal anatomy. Future investigations should extend this approach to multi-rooted teeth and more complex canal systems to further evaluate its applicability.

Taken together, the findings of the present study present the proposed optical–microscopic framework as a methodological advancement over previously reported models for electronic apex locator evaluation. Compared with extracted human teeth, acrylic blocks, and opaque resin tooth models, integrating anatomically standardized, transparent 3D-printed teeth with direct optical measurement enables simultaneous control of biological variability, electrical compatibility, and visual observability (1, 2, 11). Importantly, this framework addresses a persistent gap in the literature by allowing objective assessment of dynamic EAL functions under reproducible conditions. From an educational perspective, the capacity to directly visualize file position and movement in relation to electronic feedback offers a pedagogically valuable tool for preclinical education and training. While not intended to replace clinical evaluation, the framework provides a reproducible platform aligned with current demands for methodological rigor and transparency in dental research (23).

### Future directions and broader applicability

The experimental framework described in this study may be expanded in several directions. Variations in apical diameter, canal curvature, and resin composition could be systematically evaluated to explore their influence on EAL performance. The framework may be adapted for comparative evaluation of different impedance-based endodontic technologies beyond EAL-integrated motors.

From a broader perspective, this approach aligns with the growing emphasis on reproducibility, transparency, and methodological validation in biomedical research. By prioritizing standardized measurements and experimental controls, the framework contributes to the development of robust preclinical testing platforms that bridge the gap between laboratory investigation and clinical application.

## Conclusions

Within the limitations of this *in vitro* study, the proposed optical-microscopic framework provides a reproducible and objective method for evaluating EAL-integrated endodontic motors. By combining the transparent resin tooth model with direct physical measurement, this approach enables assessment of static and dynamic root canal length determination and offers a suitable platform for preclinical research and device evaluation.

## Data Availability

The original contributions presented in the study are included in the article/Supplementary Material, further inquiries can be directed to the corresponding author.
